# Enhancing ESG learning outcomes through gamification: An experimental study

**DOI:** 10.1371/journal.pone.0303259

**Published:** 2024-05-15

**Authors:** Fang Zhang

**Affiliations:** Center of Smart Campus Construction, Central University of Finance and Economics, Beijing, 100081, China; University of Zagreb Faculty of Electrical Engineering and Computing: Sveuciliste u Zagrebu Fakultet Elektrotehnike i Racunarstva, CROATIA

## Abstract

This study investigates the effectiveness of gamification in enhancing learning outcomes in Environmental, Social, and Governance (ESG) education. Employing a cluster randomized experiment, the research involved 22 classes from four universities, divided into gamified and traditional teaching groups. The gamified group engaged with ESG concepts through interactive, game-like elements, while the control group followed standard educational practices. The study aimed to determine whether gamification could improve ESG course effectiveness and enhance Psychological Ownership and Perceived Importance, thereby influencing learning outcomes. Data collected through post-experiment surveys were analyzed using multiple linear regression and Structural Equation Modeling (SEM). Results indicated that students in the gamified group performed significantly better in ESG exams compared to the control group, demonstrating the effectiveness of gamification in enhancing academic achievement. The SEM analysis further revealed that gamification positively impacted Psychological Ownership and Perceived Importance, which in turn significantly improved academic performance. These findings suggest that incorporating gamification in ESG education can effectively engage students and deepen their understanding of complex sustainability issues. This study contributes to the field by highlighting the potential of gamification as a transformative tool in higher education, particularly in teaching abstract and multifaceted subjects like ESG.

## Introduction

In recent years, gamification has emerged as a transformative tool in educational settings, reshaping the traditional landscapes of learning [1]. This approach, which incorporates game design elements into non-game contexts, aims to enhance engagement, motivation, and participation in learning activities [2]. Its adoption signals a paradigm shift in educational methodologies, moving away from conventional rote learning to a more interactive, student-centered approach. The core philosophy of gamification in education is rooted in the belief that the engaging and interactive nature of games can be leveraged to create more effective and immersive learning experiences.

The effectiveness of gamification in education is grounded in its ability to foster increased engagement and motivation among learners [3]. By incorporating elements such as point scoring, competition, and rules of play, educational programs become more than just repositories of knowledge—they transform into dynamic environments that actively involve students in the learning process [4,5]. This involvement is not just superficial; it taps into the inherent psychological need for accomplishment, recognition, and reward, making the learning process both enjoyable and impactful.

Recognizing its potential, numerous educational programs across various disciplines have begun integrating gamified elements into their course designs. This trend is evident not only in primary and secondary education but also in higher education and professional training. The integration of gamification strategies varies, ranging from simple point-based reward systems to intricate game-based learning modules. These adaptations are reflective of a growing understanding that different educational contexts require unique gamification approaches to maximize learner engagement and knowledge retention.

In the realm of Environmental, Social, and Governance (ESG) education, gamification holds particular promise. ESG principles, which focus on sustainable and ethical practices in business and society, are complex and multifaceted [6,7]. Traditional teaching methods often struggle to convey the full breadth and depth of these concepts in an engaging manner. Gamification, with its ability to simulate real-world scenarios and encourage interactive learning, offers a novel way to bring ESG principles to life. By doing so, it not only enhances understanding but also fosters a deeper appreciation and commitment to sustainable practices.

Despite the apparent advantages of gamified learning in ESG education, there is a notable lack of empirical research in this area. Most existing studies on gamification in education focus on general learning outcomes or specific subjects like mathematics and science [8,9]. There is a significant research gap concerning the application and impact of gamification specifically in ESG education. This gap points to the need for focused studies that explore how gamified learning can be effectively employed to teach ESG principles and what impact it has on student learning outcomes.

Addressing this research gap, the present study adopts a two-stage experimental approach. The first stage involves a randomized group experiment conducted across 22 classrooms. In this phase, one group of students experienced a gamified approach to ESG education, while a control group continued with traditional teaching methods. The aim was to assess the differential impact on students’ ESG exam scores. The second stage involved a follow-up within the gamified group, using the "Affordances-Psychological Outcomes-Perceived Importance" framework to analyze the mechanisms through which gamification affects learning outcomes. This comprehensive approach allows for a nuanced understanding of both the efficacy and the underlying processes of gamified learning in ESG education.

The study is primarily aimed at answering two critical research questions to fill the existing gap in literature: (1) Can gamified classrooms enhance the effectiveness of ESG education? (2) Does gamification in the classroom improve Psychological Ownership and Perceived Importance, and what are the mediating effects of these factors on ESG learning outcomes? By addressing these questions, the study seeks to contribute to the broader discourse on gamification in education, with a specific focus on its application and impact in teaching ESG principles. The findings are expected to offer valuable insights for educators, curriculum designers, and policymakers interested in leveraging gamification for more effective ESG education.

## Literature review

### Gamification impact on classroom effectiveness

Gamification in educational contexts is a rapidly evolving field, marked by the integration of game mechanics into learning environments to boost student engagement, motivation, and participation [10,11]. Central to this concept is the idea of ’gamification affordances,’ which refers to the characteristics of a gamified system that enable specific user interactions. These affordances are designed to transform the learning experience into a more game-like and engaging activity, leveraging elements such as points, badges, leaderboards, and narratives [12–14]. This approach has been theorized to facilitate deeper cognitive processing, enhance motivation, and promote a more active and participatory form of learning.

Empirical studies in the field of educational gamification have consistently demonstrated its positive impact on classroom effectiveness. Research indicates that gamification can lead to improved student engagement, higher motivation levels, and better academic performance. For instance, a meta-analysis found that gamified learning environments significantly improved students’ grades and engagement levels compared to traditional teaching methods [15]. These findings suggest that the integration of game elements in educational settings can create a more stimulating and effective learning environment.

### Impact on psychological ownership and perceived importance

Psychological Ownership in the context of education refers to a student’s sense of personal connection and investment in their learning process. Gamification can significantly enhance this feeling of ownership by allowing students to control certain aspects of their learning journey, such as choosing tasks or setting personal goals. When students feel a sense of ownership over their learning, they are more likely to be engaged and motivated, leading to better learning outcomes [16,17]. Gamification strategies that offer autonomy and personalized learning paths are particularly effective in fostering this sense of ownership.

The concept of Perceived Importance in education relates to the degree to which students recognize the value and relevance of what they are learning. Gamification can enhance the perceived importance of course content by presenting it in a context that is meaningful and engaging for students [18]. For example, narrative elements and real-world simulations in gamified courses can help students understand the practical applications and significance of the material [19–21]. This enhanced perception of importance is likely to result in greater attention, deeper processing of information, and better retention.

### Social influence and self-efficacy as defining elements

Psychological ownership in the educational context encompasses feelings of possession and a personal stake in the learning process. This concept is closely linked to two main factors: social influence and self-efficacy. Social influence pertains to the ways in which social interactions and observations shape one’s learning experiences [22]. Self-efficacy, on the other hand, refers to a learner’s belief in their capability to successfully engage with and complete learning tasks [23]. Both factors are crucial in fostering a sense of ownership, as they contribute to a student’s confidence and engagement in the learning process.

The impact of Psychological Ownership on learning outcomes is profound. When students feel a sense of ownership over their learning, they are more likely to be engaged, take initiative, and exhibit persistence in the face of challenges. Research has shown that high levels of psychological ownership are associated with enhanced academic performance, greater satisfaction with the learning experience, and higher levels of intrinsic motivation [24–28]. This highlights the importance of cultivating a sense of ownership in students to achieve optimal educational outcomes.

### Research model

Autonomy support in the context of gamification refers to the degree to which a learning environment allows students to exercise choice and control over their learning activities. The hypothesis H1 posits that autonomy support positively influences Psychological Ownership. This hypothesis is grounded in Self-Determination Theory, which suggests that when learners feel a sense of autonomy, they experience higher motivation and engagement. Autonomy in gamified learning can take various forms, such as choosing avatars, setting personal goals, or selecting tasks, all of which contribute to a greater sense of ownership and engagement.

**Fig 1:**
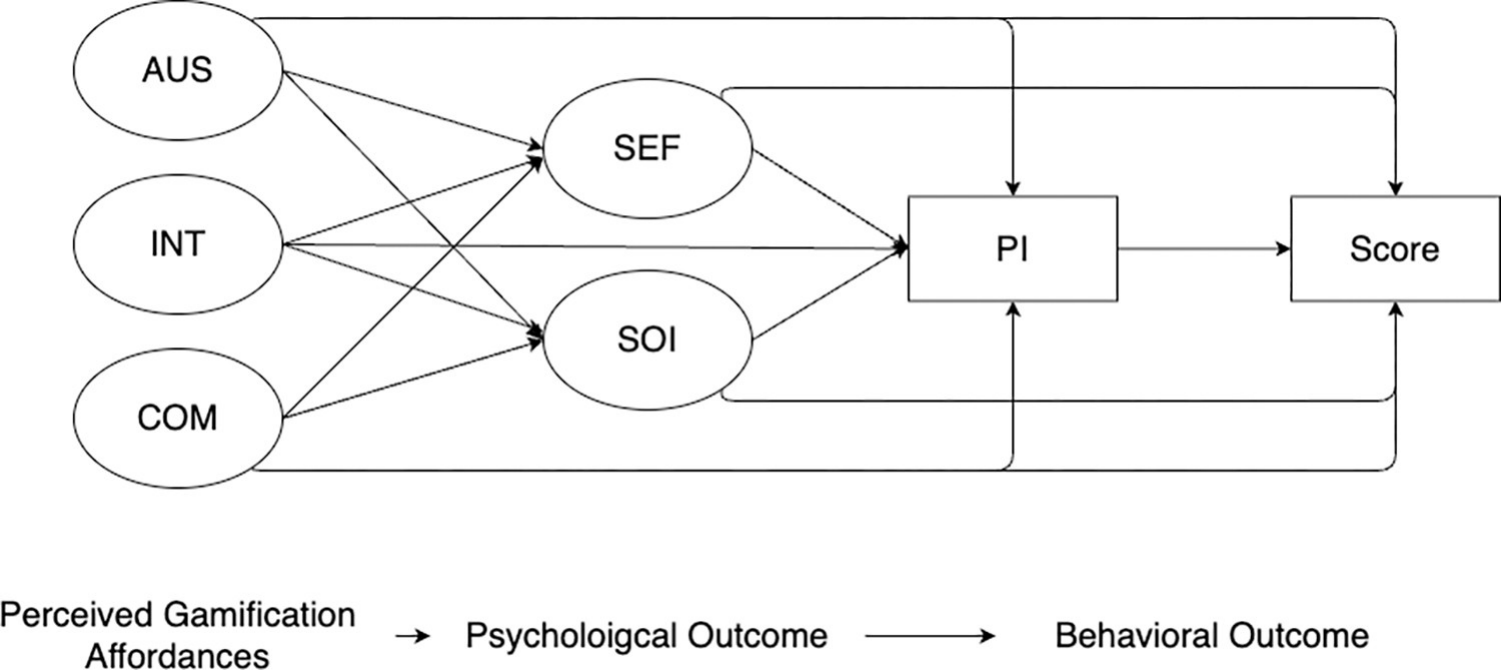
Theoretical model. Autonomy Support (AUS), Interactivity (INT), Competition (COM), Social influence (SOI), Self-efficacy (SEF), Perceived Importance (PI), Psychological Ownership (PO).

Interactivity and competition are key elements in gamified learning environments. Hypothesis H2 and H3 suggest that both interactivity and competition positively impact Psychological Ownership. Interactivity, which involves active participation and collaboration with peers, enhances the learning experience by making it more engaging and meaningful. Competition, when used constructively, can motivate students to strive for excellence and take greater interest in their learning tasks. Additionally, these elements are hypothesized (H4, H5, and H6) to influence Perceived Importance, as they create a more immersive and relevant learning experience, thereby enhancing the perceived value of the educational content. The theoretical hypothetical model is in Fig 1.

## Methods

### Experimental design

The study employed a cluster randomized experiment involving 22 classes from four universities with similar academic standings, each offering an Environmental, Social, and Governance (ESG) course in Mar 2023. The research was approved by the IRB of Central University of Finance and Economics with code and inform consent was obtained from subjects involved.

First, we selected four universities that offer ESG courses based on the admission scores of the college entrance examination. The average scores of majors offering ESG courses in these four universities are similar. Secondly, six classes were selected from the majors offered by the four universities based on the total entrance scores of the students as the sampling weight. Finally, two classes were deleted based on the adjustment of teaching intensity, leaving a total of 22 classes. Such sampling standards can meet the requirements of p value 0.05 and statistical power 0.8 [29]. The classes were randomly assigned to either the intervention group, which experienced gamified ESG education, or the control group, which continued with traditional teaching methods. The intervention group’s classroom interactions and assignments were gamified, incorporating elements like simulated business management tasks based on ESG principles. This setup provided a unique opportunity to compare the effectiveness of traditional and gamified approaches in teaching complex ESG concepts.

**Fig 2:**
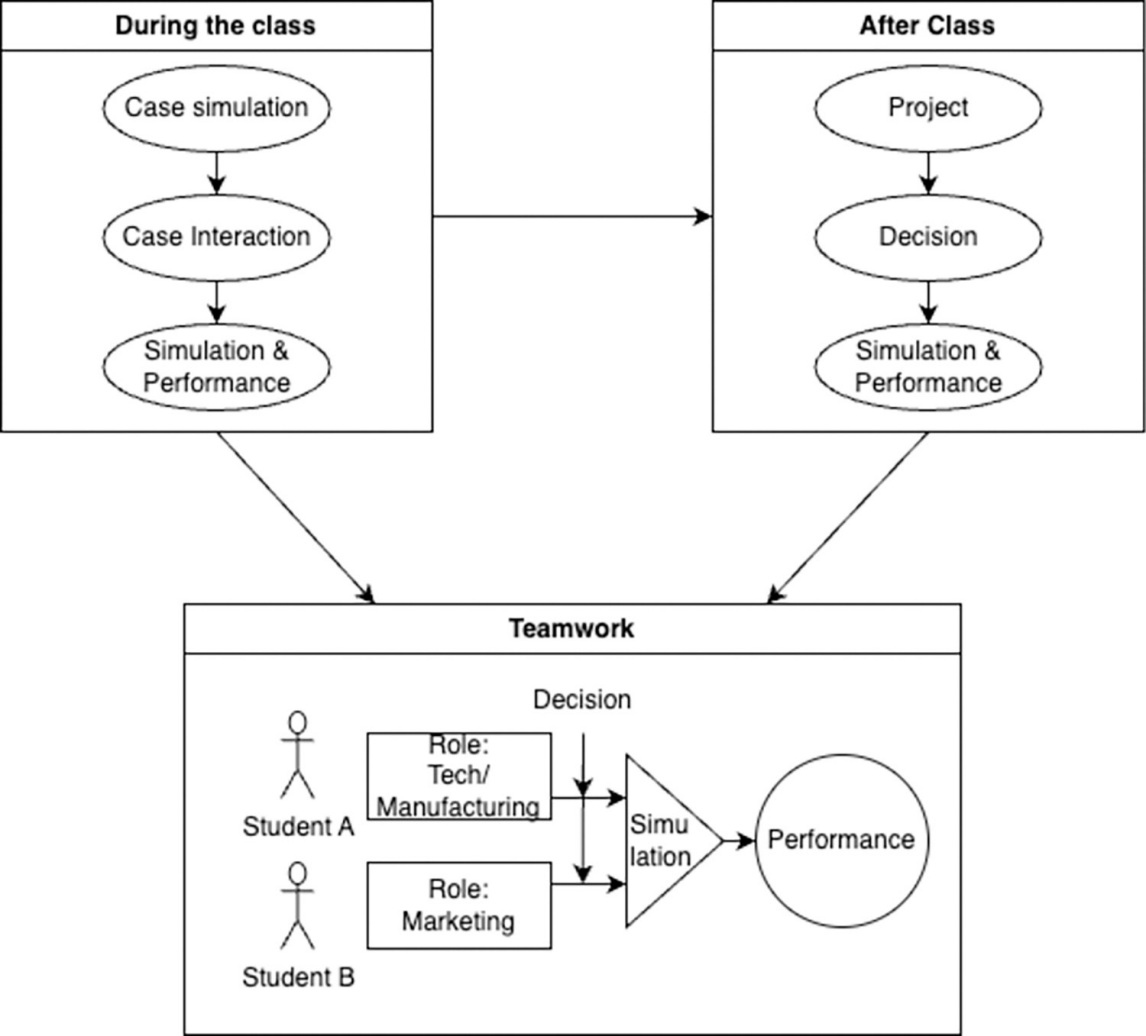
The model of gamification intervention during the ESG class.

In the intervention group, gamification was implemented through a series of interactive activities and assignments. Each student was assigned a simulated business, which they were responsible for managing according to ESG principles learned in the course. This hands-on approach allowed students to apply theoretical knowledge in a practical, engaging context(detailed in Fig 2). The gamification elements included point scoring based on ESG compliance, leaderboards to encourage healthy competition, and badges for achieving specific milestones. These elements were designed to enhance student engagement, motivation, and understanding of ESG concepts.

### Survey design

The survey [30–35] (detailed questionnaire in Appendix Table 1 in S1 File), distributed post-experiment at the end of Jun 2023, was designed to capture data on various constructs relevant to the study. These constructs included Perceived Gamification Affordance, Autonomy Support, Interactivity, Self-expression, Competition, Psychological Ownership, Self-efficacy, and Social Influence. The items for each construct were carefully selected and adapted from established scales in previous literature, ensuring their validity and relevance to the study’s context. For instance, items measuring Autonomy Support were derived from scales used in Deci and Ryan’s studies on Self-Determination Theory, while those for Psychological Ownership were adapted from Avey et al. (2009).

The survey was structured to include multiple items for each construct, using a Likert scale ranging from strongly disagree to strongly agree. This structure allowed for a nuanced capture of students’ perceptions and experiences regarding the gamified ESG course. The survey was administered electronically to ensure convenience and increase response rates. Confidentiality and anonymity were assured to all participants to encourage honest and unbiased responses. The collected data provided a rich source of information for analyzing the impact of gamification on ESG education.

### Statistical method

#### Study 1: Examining intervention effects

In Study 1, the primary objective was to assess the effectiveness of the gamified intervention on ESG course outcomes. To ensure the comparability of the intervention and control groups, a balance check was conducted on key demographic and academic variables using t-tests for continuous variables including age and household income, chi-square tests for gender. After confirming the balance between groups, multiple linear regression analysis was employed to examine the impact of the gamification intervention on students’ ESG exam scores. The regression model included the intervention condition (dummy coded: 1 = intervention group, 0 = control group) as the primary predictor variable, while controlling for relevant covariates (e.g., prior academic performance, demographic characteristics) to account for potential confounding factors. Considering the cluster design, the cluster robust standard errors were used for statistical test for coefficients.

#### Study 2: Path analysis using structural equation modeling

In Study 2, path analysis using structural equation modeling (SEM) was employed to explore the mechanisms through which gamification influenced ESG learning outcomes. Specifically, the path analysis followed these steps:

First, the structural model was evaluated by examining the hypothesized paths among the latent variables. The path coefficients, representing the direct effects, were estimated using maximum likelihood estimation. The overall fit of the structural model was assessed using various fit indices, such as the chi-square/df statistic, comparative fit index (CFI), the root mean square error of approximation (RMSEA), and the standardized root mean square residual (SRMR). Model fit was evaluated using multiple fit indices and their respective cutoff criteria [36]: the chi-square/df statistic less than 3 value indicating good fit; the CFI with values larger than .95 considered a good fit; the RMSEA with values smaller than .06 indicating a good fit; and the SRMR with values smaller than .08 suggesting acceptable fit.

Second, mediation analyses were conducted to test whether psychological ownership and perceived importance mediated the relationship between gamification affordances and academic performance. Bootstrapping techniques were employed to estimate the indirect effects and associated confidence intervals.

Finally, the final model’s path coefficients, direct and indirect effects, and model fit indices were interpreted and reported, providing insights into the mechanisms through which gamification influenced ESG learning outcomes and the strength of these relationships. The path analysis using SEM allowed for the simultaneous examination of multiple relationships, accounting for measurement errors and testing complex mediational processes

## Result

### Descriptive analysis

The descriptive analysis (See Table 1) focused on comparing the control and treatment groups across several demographic variables such as age, sex, and household income. This comparison was crucial to establish baseline equivalence between the two groups. The results indicated no significant differences in these demographic factors, with both groups showing similar distributions in age, gender composition (approximately 80% of females), and household income levels. These findings suggest that the two groups were comparable at the outset of the experiment, providing a solid foundation for assessing the impact of the gamification intervention.

**Table 1:** Descriptive statistics and sample balance test.

	Control	Treatment	p-value
Numbers of Participants	440	438	-
Gender (mean (SD))	0.80 (0.40)	0.80 (0.40)	0.973
Age (mean (SD))	23.97 (0.82)	24.01 (0.83)	0.463
Household Income (mean (SD))	2.99 (1.39)	3.06 (1.42)	0.471

Additionally, a comparison of baseline academic performance was conducted to ensure that any observed differences in post-intervention outcomes could be attributed to the gamification treatment. The analysis revealed no significant differences in the initial ESG course performance between the control and intervention groups. This similarity in baseline academic performance further reinforced the validity of the subsequent comparative analysis of the intervention’s effects.

### Result of study 1: Effectiveness of the gamification intervention

In Study 1, the primary outcome of interest was the students’ performance on the ESG exam. The results (See Table 2) from the multivariate linear regression analysis revealed a statistically significant positive effect of the gamification intervention. Students in the gamified group scored, on average, approximately 19 points higher than those in the control group, controlling for other demographic factors. This finding was robust across various model specifications, indicating a strong and consistent effect of gamification on improving ESG exam scores.

**Table 2:** Regression for testing gamification treatment effect.

	Score(%)	
Group	18.956***	19.009***
	(1.869)	(1.872)
Gender		0.521
		(2.337)
Age		0.144
		(1.134)
Household Income		-0.861
		(0.666)
Constant	50.183***	48.899*
	(1.320)	(27.363)
Observations	878	878
R2	0.105	0.107

*Note*: The multilevel structure of each cluster was considered, and the standard error is cluster robust error clustered by class.

*p<0.10

**p<0.05

***p<0.01.

The analysis also considered the impact of demographic variables such as sex, age, and household income on exam scores. While these factors were controlled for in the regression models, they did not significantly influence the relationship between gamification and academic performance. This result suggests that the positive effect of gamification on ESG learning outcomes was not confounded by these demographic characteristics.

### Result of study 2: Path analysis and mechanisms of gamification

In Study 2, the Structural Equation Modeling (SEM) analysis provided insights into the mechanisms through which gamification influenced learning outcomes (See Table 3). The model demonstrated a good fit with the data, indicating that the hypothesized relationships were consistent with the observed patterns. The analysis revealed that gamification affordances significantly influenced Psychological Ownership and Perceived Importance, which in turn had a positive impact on students’ academic performance in the ESG course.

**Table 3:** Path analysis for gamification effects.

	Estimate	Std.Err	z-value	P(>|z|)
SOI ~				
AUS	0.415	0.064	6.476	0.000
INT	0.231	0.057	4.055	0.000
COM	0.426	0.080	5.319	0.000
SEF ~				
AUS	0.268	0.053	5.018	0.000
INT	0.352	0.060	5.887	0.000
COM	0.385	0.074	5.207	0.000
PI ~				
SOI	0.424	0.116	3.665	0.000
SEF	0.588	0.138	4.263	0.000
AUS	0.030	0.080	0.374	0.708
INT	0.034	0.076	0.450	0.653
COM	-0.009	0.099	-0.090	0.928
Score ~				
PI	23.534	1.145	20.560	0.000
SOI	3.680	1.984	1.855	0.064
SEF	1.898	2.256	0.842	0.400
AUS	0.368	1.262	0.291	0.771
INT	0.393	1.178	0.333	0.739
COM	0.078	1.573	0.049	0.961

*Note*: Autonomy Support (AUS), Interactivity (INT), Competition (COM), Social influence (SOI), Self-efficacy (SEF), Perceived Importance (PI). The multilevel structure of each cluster was considered, and the standard error is cluster robust error clustered by class.

The SEM results highlighted several key pathways. Autonomy support, interactivity, and competition (as elements of gamification affordances) all positively influenced Psychological Ownership. Furthermore, Psychological Ownership was found to have a significant positive effect on Perceived Importance, validating the hypothesized mediating role of these constructs. Finally, both Psychological Ownership and Perceived Importance were significant predictors of improved academic performance, illustrating their critical role in the efficacy of gamified ESG education.

The hypothesized structural equation model was evaluated for overall fit using several well-established fit indices. The model demonstrated an excellent fit to the data, with a chi-square/df value less than 3 (χ2/df = 1.256), indicating that the model was consistent with the sample data. The comparative fit index (CFI = .988) exceeded the recommended cutoff of .95 for good fit, further supporting the model’s acceptable fit. Additionally, the root mean square error of approximation (RMSEA = .024) was well below the .06 threshold, suggesting a close fit between the hypothesized model and the population data (Steiger & Lind, 1980). Finally, the standardized root mean square residual (SRMR = .032) was substantially lower than the .08 cutoff, indicating minimal discrepancies between the observed and model-implied covariance matrices. Collectively, these fit indices provided strong evidence for the tenability of the hypothesized structural equation model in explaining the relationships among the latent variables of interest.

The results of this study provide compelling evidence for the effectiveness of gamification in enhancing ESG learning outcomes. The significant improvement in exam scores among students in the gamified group, coupled with the SEM analysis, underscores the value of incorporating game-like elements into educational settings. These findings contribute to the growing body of literature supporting gamification as a viable and impactful educational strategy, particularly in the context of complex and multifaceted subjects like ESG.

## Discussion

The primary objective of this study was to explore the effectiveness of gamification in ESG education and to understand the underlying mechanisms through which it influences learning outcomes. This was addressed through two key research questions: firstly, whether gamified classrooms can enhance the effectiveness of ESG courses, and secondly, the role of Psychological Ownership and Perceived Importance as mediators in this process. The results from both experimental and survey analyses provided valuable insights, contributing significantly to the existing body of knowledge on educational gamification.

The study successfully achieved its goals by demonstrating the positive impact of gamification on ESG course outcomes and elucidating the psychological processes involved. The findings not only affirmed the potential of gamification as an effective pedagogical tool in higher education but also shed light on the importance of Psychological Ownership and Perceived Importance in the context of gamified learning environments.

### Main findings

The study’s results confirmed several key hypotheses. The significant improvement in exam scores among students in the gamified group supported the hypothesis that gamification enhances classroom effectiveness. Additionally, the SEM analysis validated the proposed relationships between gamification affordances, Psychological Ownership, Perceived Importance, and academic performance, thereby confirming the mediating role of these psychological constructs.

The findings align well with existing theories in educational psychology and gamification research. The positive impact of gamification on learning outcomes resonates with the principles of Self-Determination Theory and the concept of active learning. However, some discrepancies were noted, particularly in the magnitude of impact attributed to specific gamification elements like competition and interactivity. These variations offer new avenues for future research to explore the nuanced effects of different gamification strategies.

The positive effect of the gamification intervention on students’ ESG exam scores aligns with numerous prior studies that have demonstrated the beneficial impact of gamification on academic performance across various subjects [37–40]. This corroborates the notion that incorporating game-like elements can create an engaging and immersive learning environment, facilitating better knowledge acquisition and retention.

Furthermore, the study’s findings regarding the mediating roles of Psychological Ownership and Perceived Importance resonate with established theories in educational psychology. The positive influence of gamification affordances on these psychological constructs is consistent with the principles of Self-Determination Theory [41] and the concept of active learning [42]. By fostering autonomy, interactivity, and healthy competition, gamified learning environments can enhance students’ sense of ownership and perceived value of the educational content, ultimately leading to improved learning outcomes.

### Theoretical contribution

This study makes significant theoretical contributions, particularly in the context of gamifying complex subjects like ESG. It extends the application of gamification theory beyond its traditional domains, demonstrating its effectiveness in fostering a deeper understanding of multifaceted and abstract concepts. Moreover, by integrating psychological constructs such as Psychological Ownership and Perceived Importance into the gamification framework, the study enriches the theoretical underpinnings of educational gamification [43–45].

By demonstrating the effectiveness of gamification in improving learning outcomes for a complex subject like Environmental, Social, and Governance (ESG), this research extends the application of gamification theory beyond its traditional domains. Existing gamification literature has primarily focused on subjects such as math, science, and language learning. However, this study provides empirical evidence that gamification can also be a powerful pedagogical tool for teaching multifaceted and abstract concepts related to sustainability and ethical business practices. This broadens the scope of gamification theory and opens up new avenues for its application in diverse academic disciplines.

A significant theoretical contribution of this study lies in the integration of the psychological constructs of Psychological Ownership and Perceived Importance into the gamification framework. While previous research has explored the impact of gamification on motivation and engagement, this study sheds light on the crucial roles played by these specific psychological mechanisms.

The findings suggest that gamification affordances, such as autonomy support, interactivity, and competition, can foster a sense of Psychological Ownership among students. This feeling of personal investment and connection with the learning process, in turn, enhances the Perceived Importance of the educational content. By establishing these relationships, the study enriches our theoretical understanding of how gamification influences cognitive and affective processes, leading to improved learning outcomes.

The findings suggest that gamification affordances, such as autonomy support, interactivity, and competition, can foster a sense of Psychological Ownership among students. This feeling of personal investment and connection with the learning process, in turn, enhances the Perceived Importance of the educational content. By establishing these relationships, the study enriches our theoretical understanding of how gamification influences cognitive and affective processes, leading to improved learning outcomes.

The positive influence of gamification affordances on Psychological Ownership and Perceived Importance aligns with the principles of SDT [41]. SDT posits that individuals have innate psychological needs for autonomy, competence, and relatedness, and when these needs are satisfied, they experience increased motivation and engagement [46–48]. The gamification elements of autonomy support, interactivity (fostering relatedness), [49]and competition (enhancing competence) can potentially fulfill these psychological needs, thereby fostering a sense of ownership [30] and perceived importance among students [47].

By corroborating the tenets of SDT in the context of gamified learning environments, this study reinforces the theoretical underpinnings of gamification and contributes to a deeper understanding of the psychological mechanisms at play.

The study’s findings also contribute to the theoretical domain of active learning. Active learning approaches emphasize the importance of student engagement, participation, and hands-on experiences in the learning process. Gamification, with its interactive and immersive elements, aligns well with the principles of active learning. By demonstrating the positive impact of gamification on Psychological Ownership and Perceived Importance, this research provides empirical support for the effectiveness of active learning strategies [50–52] in fostering a sense of personal investment and perceived relevance among students.

These theoretical implications not only validate existing theories but also extend their application to the gamification context [53,54], offering new insights and avenues for further exploration.

### Practical implication

The practical implications of this research are far-reaching, particularly for educators and curriculum designers in the realm of ESG and sustainable development. The demonstrated effectiveness of gamification in improving ESG learning outcomes suggests that educational institutions should consider integrating gamified elements into their curricula. This approach could play a crucial role in preparing students to tackle the complex challenges of sustainability and ethical governance in their professional careers.

The demonstrated effectiveness of gamification in improving ESG learning outcomes suggests that educational institutions should strongly consider integrating gamified elements into their curricula. Curriculum designers can leverage the study’s insights to develop immersive and engaging learning experiences that incorporate game-like mechanics, such as simulations, challenges, and interactive scenarios.

For instance, in the context of ESG education, students could be tasked with managing a simulated business, where they must make strategic decisions that align with ESG principles. This hands-on approach not only reinforces theoretical knowledge but also allows students to experience the practical applications and consequences of their choices in a risk-free environment.

Successful implementation of gamified learning experiences requires faculty members to be adequately trained and supported. Professional development programs can be designed to equip educators with the necessary skills and knowledge to effectively design and facilitate gamified lessons. These programs should cover topics such as game mechanics, instructional design principles, and strategies for fostering engagement and motivation through gamification.

Additionally, institutions should provide ongoing support and resources to faculty members, enabling them to stay up-to-date with the latest developments in gamification and educational technology. This can include access to online communities, workshops, and collaborative platforms for sharing best practices and innovative approaches.

At the institutional and governmental levels, policymakers play a crucial role in promoting and supporting the adoption of gamified learning approaches. This may involve allocating dedicated funding and resources for the development and implementation of gamified curricula, as well as investing in the necessary infrastructure and technology.

Furthermore, policymakers can establish guidelines and standards for the effective integration of gamification in education, ensuring consistency and quality across institutions. These guidelines should be informed by research findings, such as those from this study, to ensure that gamification strategies are grounded in sound theoretical and empirical foundations.

Effective implementation of gamification in education can be further facilitated by fostering collaboration and knowledge sharing among educational institutions, researchers, and industry partners. This can take the form of conferences, workshops, and online platforms where stakeholders can exchange ideas, best practices, and lessons learned from their respective experiences with gamified learning.

Industry partners, particularly those operating in sustainability-related fields, can provide valuable insights and real-world scenarios that can be incorporated into gamified ESG curricula. This collaboration can not only enhance the practical relevance of the learning experiences but also facilitate smoother transitions for students entering the workforce.

By considering these practical implications and taking concrete steps towards implementing gamified learning approaches, educational institutions and policymakers can play a pivotal role in shaping the future of ESG education and preparing students to tackle complex sustainability challenges and ethical governance issues.

### Limitations and future research

While the study provides valuable insights, it is not without limitations. The experimental design, though robust, was limited to a specific academic context, which may affect the generalizability of the findings. Future research could replicate this study in different educational settings or with diverse student populations to validate the results. Additionally, exploring the long-term impact of gamification on student learning and engagement would provide a more comprehensive understanding of its effectiveness as a pedagogical tool.

## Conclusion

Based on the current situation where sustainability and ethical governance have become paramount concerns, and combined with Self-Determination Theory and Active Learning Theory, this article tests the hypothesis that gamification can enhance learning outcomes in Environmental, Social, and Governance (ESG) education. The experimental results support the positive impact of gamification on academic performance in ESG courses. The causal paths revealed through Structural Equation Modeling indicate that gamification affordances, such as autonomy support, interactivity, and competition, foster a sense of Psychological Ownership and Perceived Importance among students, which in turn significantly improve their learning outcomes. The findings of this article support the effectiveness of gamification as a pedagogical tool, contradict the notion that gamification is limited to certain subjects, and develop the theoretical understanding of how gamification influences cognitive and affective processes in education. Consequently, this article recommends the integration of gamified elements into ESG curricula and the provision of faculty training and institutional support to facilitate the successful implementation of gamified learning experiences. By leveraging gamification, educational institutions can better prepare students to tackle the complex challenges of sustainability and ethical governance in their future endeavors.

## Supporting information

S1 Checklist (DOCX)

S1 File (DOCX)
