# Supplementary material for: Enhancing ESG learning outcomes through gamification: An experimental study
Source: PLoS One. 2024 May 15;19(5):e0303259. doi: 10.1371/journal.pone.0303259 (PMC11095718; doi:10.1371/journal.pone.0303259)
Supplement: S1 File — (DOCX) [file pone.0303259.s002.docx]

Appendix Table 1 The construction of questionnaire used in Study 2

| Construct | Item | Reference |
| --- | --- | --- |
| **Perceived Gamification Affordance** | | Du et al. (2020);Suh, Cheung et al. (2017);Suh and Wagner (2017) |
| Autonomy Support | |  |
| AUS1 | "I can make personal decisions about my activities in this course." |  |
| AUS2 | "I have the freedom to decide how to engage with the course content." |  |
| AUS3 | "This course enhances my sense of choice and freedom in learning." |  |
| Interactivity | |  |
| INT1 | "This course facilitates interaction with my peers during learning activities." |  |
| INT2 | "I have ample opportunities to interact with others in this course." |  |
| INT3 | "The course design encourages meaningful dialogue with my peers." |  |
| Self-expression | |  |
| SEE1 | "The course allows me to express my identity through its elements." |  |
| SEE2 | "I can express myself freely in the way I prefer in this course." |  |
| SEE3 | "This course enables me to distinguish myself from others through its activities." |  |
| Competition | |  |
| COM1 | "I engage in competition with my peers in this course." |  |
| COM2 | "I have opportunities to compare my performance with that of others in the course." |  |
| COM3 | "My active participation in this course can influence the status of others." |  |
| **Psychological Ownership** | | Avey et al. (2009);Lee and Suh (2015) |
| Self-efficacy | |  |
| SEF1 | "I feel confident about contributing to the success of this gamified class." |  |
| SEF2 | "I believe I can make a positive difference in this gamified learning environment." |  |
| SEF3 | "I am confident in setting and achieving high performance goals in this class." |  |
| Social Influence | | [Venkatesh et al. (2012)](https://www.sciencedirect.com/science/article/pii/S0268401222000081#bib68) |
| SOI1 | "People important to me believe I should participate in this gamified class." |  |
| SOI2 | "Individuals who influence my behavior support my use of this gamified class." |  |
| SOI3 | "People whose opinions I value recommend that I engage with this gamified class." |  |
